# Supplementary material for: Visual timing-tuned responses in human association cortices and response dynamics in early visual cortex
Source: Nat Commun. 2022 Jul 8;13:3952. doi: 10.1038/s41467-022-31675-9 (PMC9270326; doi:10.1038/s41467-022-31675-9)
Supplement: Supplementary file 1 — Supplementary Information [file 41467_2022_31675_MOESM1_ESM.pdf]

Visual timing-tuned responses in human association cortices and response dynamics in early visual cortex

Supplementary Information

Evi Hendrikx[1+], Jacob M. Paul[1,2+], Martijn van Ackooij[1], Nathan van der Stoep[1], Ben M. Harvey[1]*

[+] Equal contribution
[1] Experimental Psychology, Helmholtz Institute, Utrecht University, Heidelberglaan 1, Utrecht 3584 CS, Netherlands
[2] Melbourne School of Psychological Sciences, University of Melbourne, Redmond Barry Building, Parkville 3010, Victoria, Australia

*Corresponding author:

Ben M. Harvey

Experimental Psychology, Helmholtz Institute, Utrecht University
Heidelberglaan 1
Utrecht 3584 CS
Netherlands
b.m.harvey@uu.nl

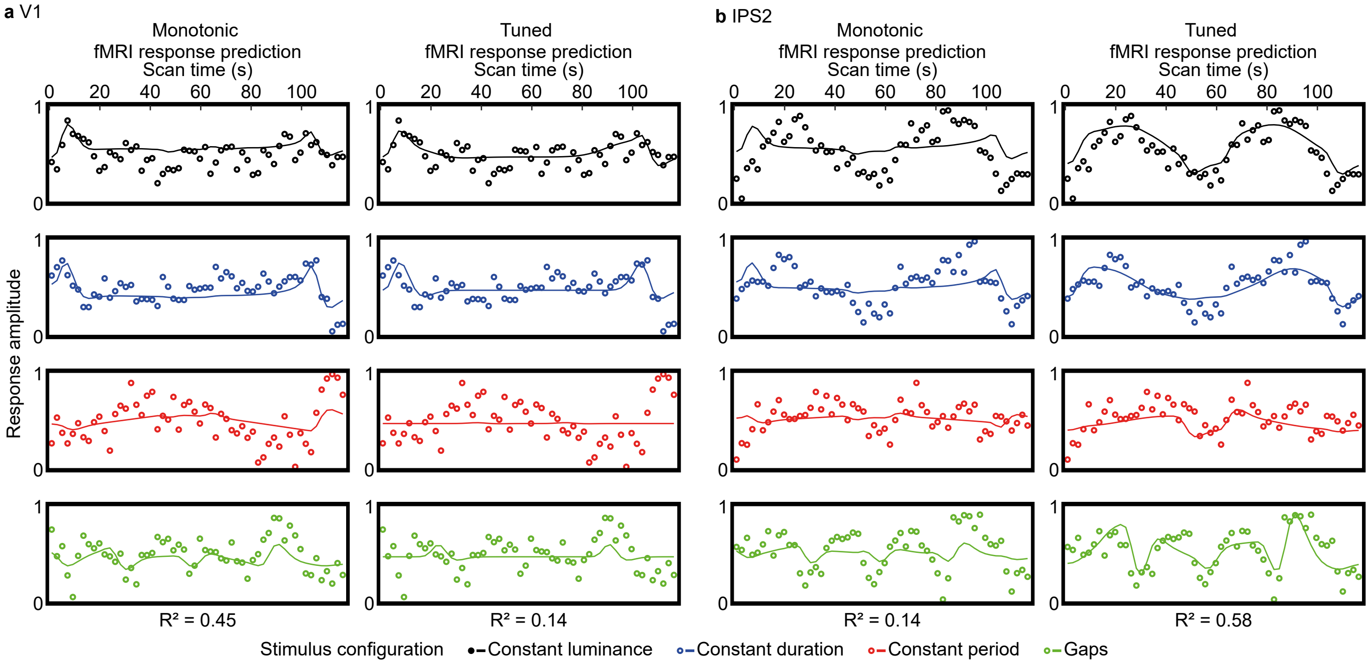

Supplementary Fig. 1 Comparison of monotonic and tuned model fits from representative voxels. Both monotonic and tuned response models were fit to the data from every voxel, and their goodness of fit compared under cross-validated to determine the best fitting response model for each voxel. **a** For a voxel in V1, the monotonic response model's predictions (lines, left panel) fit the measured responses (circles) more closely than the tuned response model's predictions (lines, right panel). **b** For a voxel in visual field map IPS2, the tuned response model's predictions (lines, right panel) fit the measured responses (circles) more closely than the monotonic response model's predictions (lines, left panel). Source data are provided as a Source Data file.

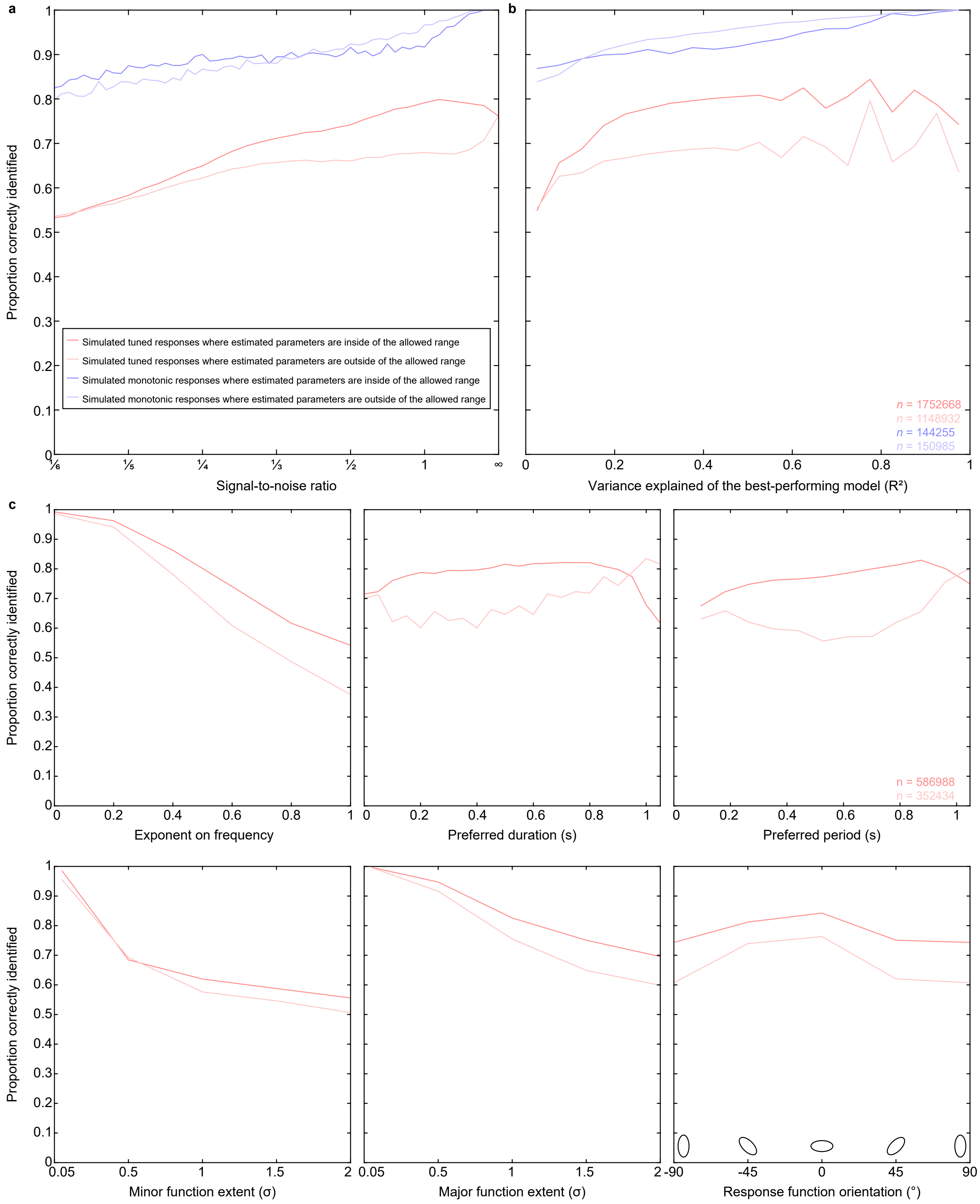

Supplementary Fig. 2: Proportion of correctly classified simulated monotonic and tuned responses during model validation. a Simulated responses following known monotonic and tuned functions were passed through our response model fitting and comparison procedures. The proportion of each simulated data set classified correctly increased when less noise was added. This proportion was higher for simulated monotonic responses. For simulated tuned responses, a higher proportion was correctly classified when

the estimated parameters in the tuned response model were inside the range allowed by our tuned response model voxel selection. At higher signal-to-noise ratios, a higher proportion of simulated monotonic responses were correctly classified when the estimated parameters in the tuned response model were outside the range allowed by our tuned response model voxel selection. In both cases, this demonstrates that our tuned response model voxel selection correctly excludes tuned responses that cannot reliably be distinguished from monotonic responses. **b** The same data ordered by the variance explained in the best-performing model in the data on which the model was fit (i.e. before cross-validation). This demonstrates that the ability to correctly classify both response types was consistent above our variance explained threshold (0.2) then dropped sharply. *n* indicates the amount of simulated data points and is identical for panel a and b. **c** Proportion of correctly classified tuned responses changes with various parameters of the underlying known response function, in all responses with a variance explained above 0.2. Some parameters reduced the ability to correctly identify tuned responses, and these parameters made the tuned response function approximate a monotonic function. Such parameters were rarely found in the fMRI responses from areas showing evidence of tuning. *n* indicates the amount of included simulated data points. Source data are provided as a Source Data file.

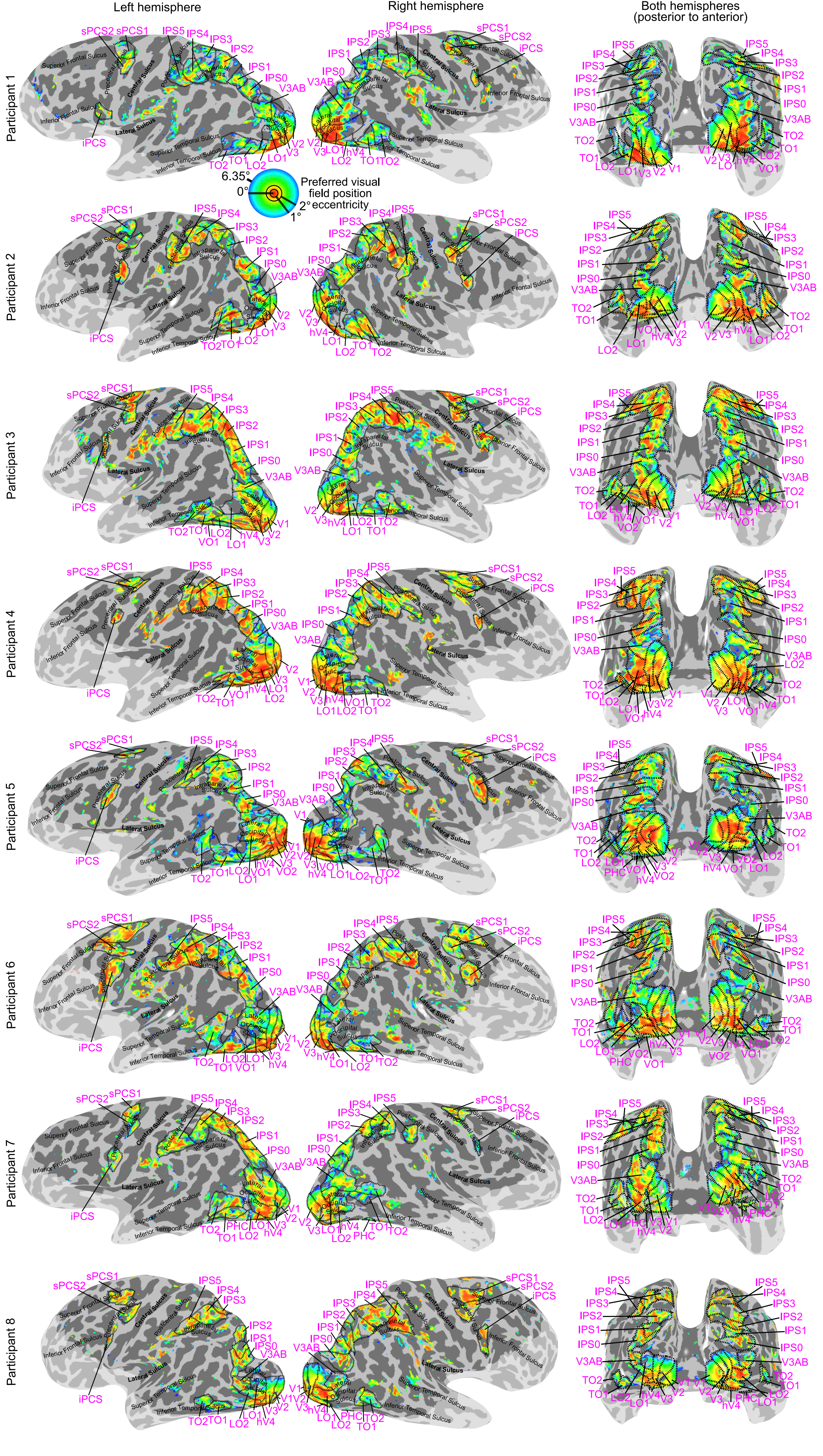

Left hemisphere
Right hemisphere
Both hemispheres
(posterior to anterior)
Participant 1
Participant 2
Participant 3
Participant 4
Participant 5
Participant 6
Participant 7
Participant 8
6.35°
0°
2°
1°
Preferred visual
field position
eccentricity

Supplementary Fig. 3 Preferred visual field position eccentricities of voxels throughout the visual field map hierarchy. Eccentricity preferences for voxels with over 0.1 variance explained by the response model. Visual field map borders are shown as black dashed lines, and named in magenta text. The light shaded region is outside the fMRI recording volume. Images adapted from Harvey, Dumoulin, Fracasso & Paul (2020, Current Biology).

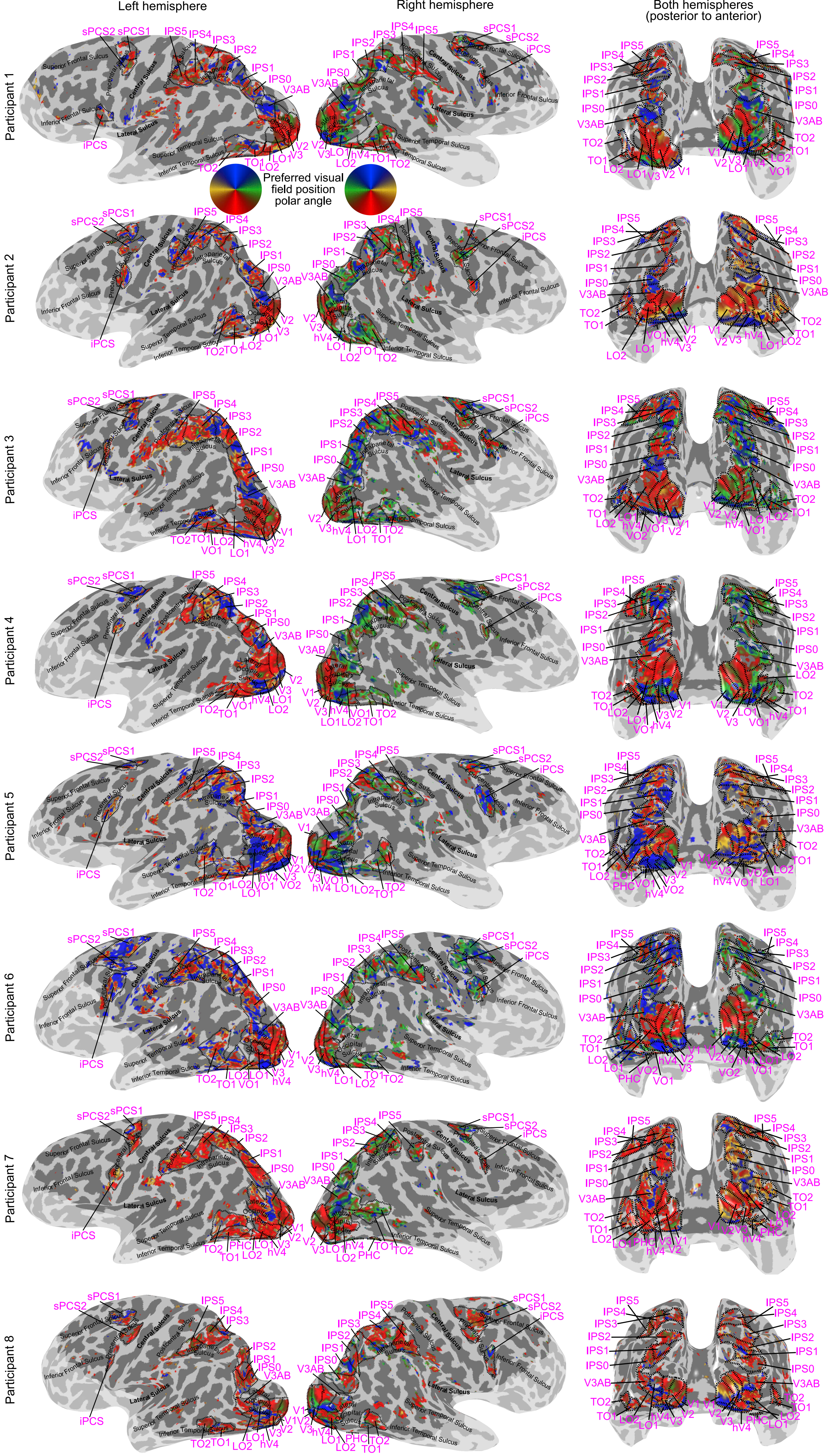

Left hemisphere
Right hemisphere
Both hemispheres
(posterior to anterior)
Preferred visual
field position
polar angle
Participant 1
Participant 2
Participant 3
Participant 4
Participant 5
Participant 6
Participant 7
Participant 8

Supplementary Fig. 4 Preferred visual field position angle of voxels throughout the visual field map hierarchy. Polar angle preferences for voxels with over 0.1 variance explained by the response model. Visual field map borders are shown as black dashed lines, and named in magenta text. The light shaded region is outside the fMRI recording volume. Images adapted from Harvey, Dumoulin, Fracasso & Paul (2020, Current Biology).

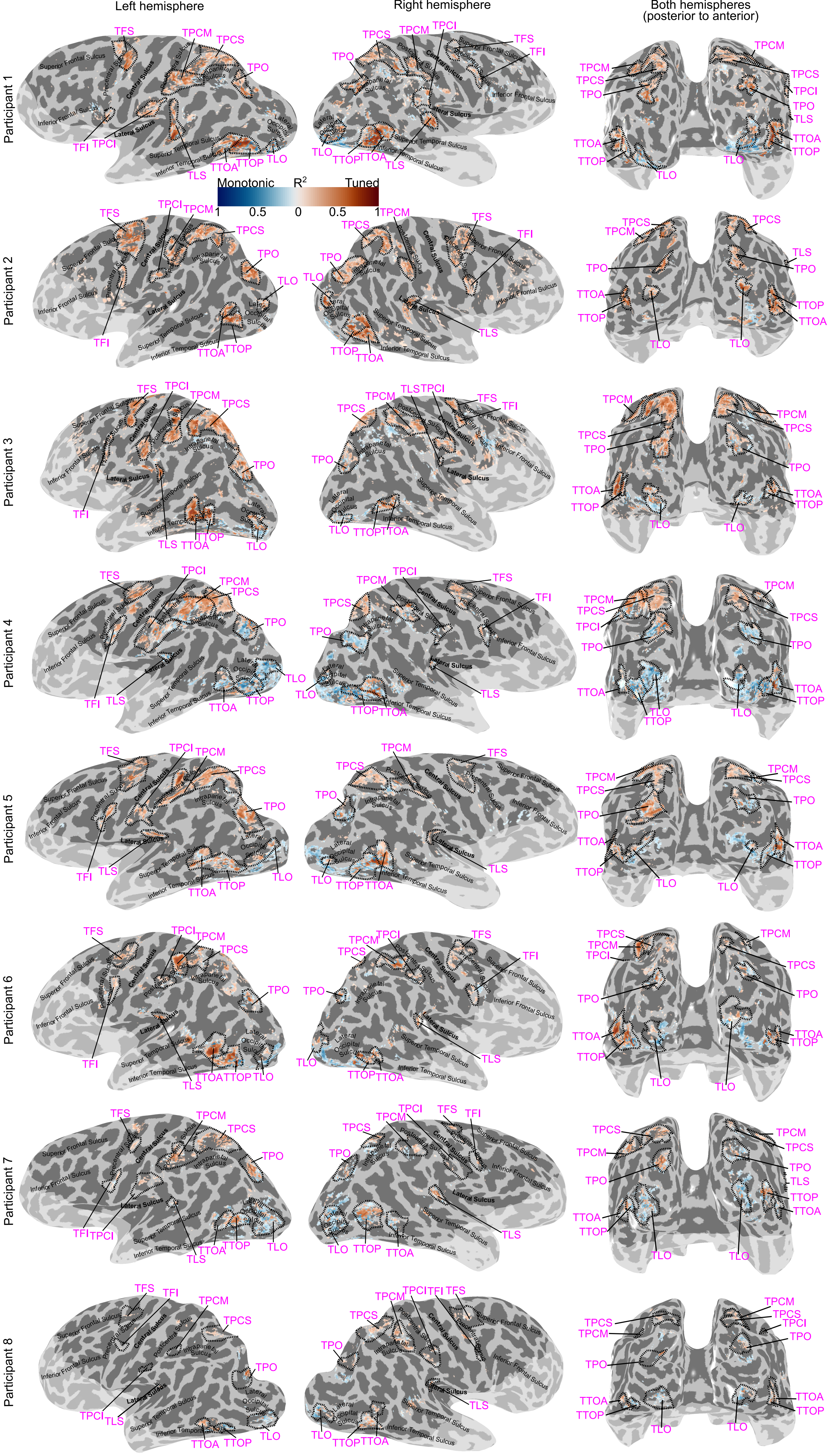

Left hemisphere
Right hemisphere
Both hemispheres
(posterior to anterior)
Participant 1
Participant 2
Participant 3
Participant 4
Participant 5
Participant 6
Participant 7
Participant 8
Monotonic
R^2
Tuned
1
0.5
0
0.5
1
TFS
TFI
TPCM
TPCS
TPCI
TPO
TLS
TLO
TTOA
TTOP
Superior Frontal Sulcus
Inferior Frontal Sulcus
Precentral Sulcus
Central Sulcus
Postcentral Sulcus
Intraparietal Sulcus
Lateral Sulcus
Superior Temporal Sulcus
Inferior Temporal Sulcus
Lateral Occipital Sulcus

Supplementary Fig. 5 Progressions of the best-performing model of responses to visual event timing. Each voxel is colored (blue or red) showing which response model (monotonic or tuned respectively) predicts recorded responses in cross-validated data best. The intensity of the color shows the variance explained by the best-performing model under cross-validation within each voxel (averaged across both cross-validation splits, for voxels with a variance explained above 0.1 for the best fitting model). Timing map borders are shown as black dashed lines, and named in magenta text. The light shaded region is outside the fMRI recording volume.

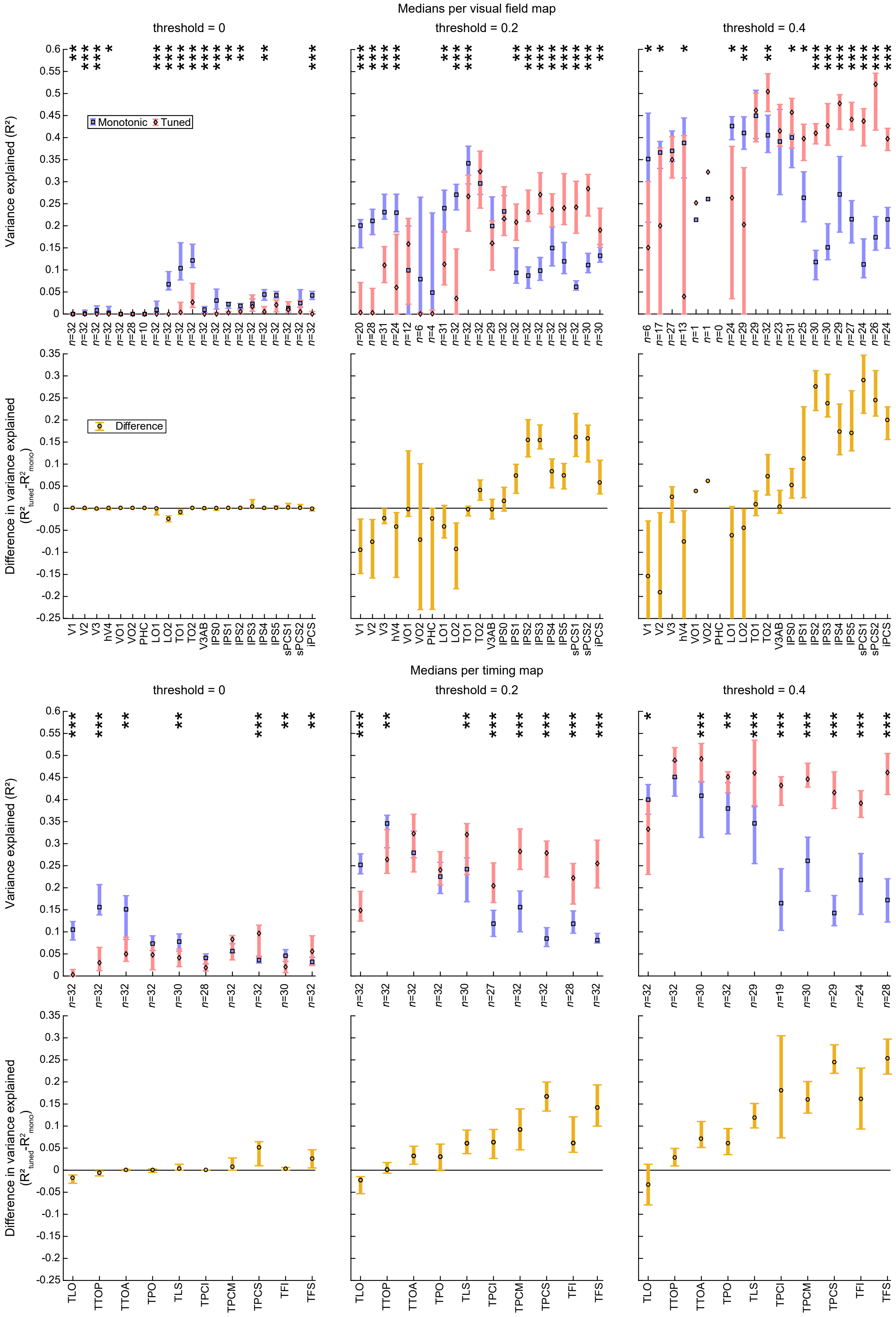

Medians per visual field map
threshold = 0
threshold = 0.2
threshold = 0.4
Variance explained (R²)
Monotonic
Tuned
Difference in variance explained (R²tuned-R²mono)
Difference
V1 V2 V3 hV4 VO1 VO2 PHC LO1 LO2 TO1 TO2 V3AB IPS0 IPS1 IPS2 IPS3 IPS4 IPS5 sPCS1 sPCS2 iPCS
Medians per timing map
threshold = 0
threshold = 0.2
threshold = 0.4
Variance explained (R²)
Difference in variance explained (R²tuned-R²mono)
TLO TTOP TTOA TPO TLS TPCI TPCM TPCS TFI TFS

Supplementary Fig. 6: Model comparisons using medians per hemisphere show similar results with a variance explained threshold above 0 for both visual field maps and timing maps. The threshold for voxel selection was a variance explained of either 0 (left column), 0.2 (middle column), or 0.4 (right column) for the best fitting model in the data on which the model was fit. A threshold of 0.2 and 0.4 result in a gradual transition from monotonic to tuned responses, starting in similar areas. The threshold of 0 deviates from this pattern because there are plenty of voxels within the maps that don't convincingly respond at all to any model. In all cases, the median value of cross-validated variance explained was taken after thresholding in each measured visual field map example. Points show the median across measured visual field map examples, error bars show 95% confidence intervals (from 1000 bootstrap iterations) and two-sided Wilcoxon signed rank tests were used in visual field maps where $n>1$.*$p < 0.05$ **$p < 0.01$, and ***$p < 0.001$, FDR corrected for multiple comparisons. *n* indicates the amount of hemispheres included in the comparison in each map. Source data are provided as a Source Data file.

Visual field map	Monotonic fit (R^2)	Tuned fit (R^2)	*Z* statistic	Effect size (*r*)	*p* value
Threshold 0					
V1	0.00 [0.00, 0.00]	0.00 [0.00, 0.00]	-3.14	-0.55	0.003
V2	0.00 [0.00, 0.01]	0.00 [0.00, 0.00]	-3.58	-0.63	8×10^{-4}
V3	0.01 [0.00, 0.02]	0.00 [0.00, 0.00]	-4.41	-0.78	4×10^{-5}
hV4	0.00 [0.00, 0.02]	0.00 [0.00, 0.00]	-2.59	-0.46	0.015
VO1	0.00 [0.00, 0.00]	0.00 [0.00, 0.00]	-0.41	-0.07	0.717
VO2	0.00 [0.00, 0.00]	0.00 [0.00, 0.00]	-0.83	-0.16	0.451
PHC	0.00 [0.00, 0.00]	0.00 [0.00, 0.00]	-0.18	-0.06	0.859
LO1	0.01 [0.00, 0.03]	0.00 [0.00, 0.00]	-4.19	-0.74	8×10^{-5}
LO2	0.07 [0.05, 0.10]	0.00 [0.00, 0.00]	-4.90	-0.87	1×10^{-5}
TO1	0.10 [0.08, 0.16]	0.00 [0.00, 0.03]	-4.94	-0.87	1×10^{-5}
TO2	0.12 [0.11, 0.16]	0.03 [0.01, 0.07]	-4.47	-0.79	4×10^{-5}
V3AB	0.01 [0.00, 0.02]	0.00 [0.00, 0.00]	-4.36	-0.77	5×10^{-5}
IPS0	0.03 [0.01, 0.06]	0.00 [0.00, 0.00]	-4.15	-0.73	9×10^{-5}
IPS1	0.02 [0.01, 0.03]	0.00 [0.00, 0.01]	-3.37	-0.59	0.002
IPS2	0.02 [0.01, 0.02]	0.01 [0.00, 0.01]	-2.82	-0.50	0.008
IPS3	0.02 [0.01, 0.03]	0.02 [0.01, 0.04]	1.40	0.25	0.211
IPS4	0.04 [0.03, 0.06]	0.01 [0.00, 0.02]	-3.03	-0.54	0.004
IPS5	0.04 [0.04, 0.05]	0.02 [0.00, 0.03]	-1.92	-0.34	0.077
sPCS1	0.01 [0.01, 0.03]	0.01 [0.00, 0.03]	0.90	0.16	0.431
sPCS2	0.02 [0.02, 0.06]	0.00 [0.00, 0.03]	-0.90	-0.16	0.431
iPCS	0.04 [0.03, 0.05]	0.00 [0.00, 0.01]	-4.68	-0.83	2×10^{-5}
Threshold 0.2					
V1	0.20 [0.15, 0.21]	0.00 [0.00, 0.07]	-3.58	-0.80	6×10^{-4}
V2	0.21 [0.18, 0.24]	0.00 [0.00, 0.06]	-4.01	-0.76	1×10^{-4}
V3	0.23 [0.22, 0.27]	0.11 [0.07, 0.15]	-4.74	-0.85	1×10^{-5}
hV4	0.23 [0.19, 0.27]	0.06 [0.00, 0.18]	-3.57	-0.73	6×10^{-4}
VO1	0.10 [0.00, 0.20]	0.16 [0.02, 0.22]	0.00	-0.00	1
VO2	0.08 [0.00, 0.26]	0.00 [0.00, 0.00]	-1.15	-0.47	0.290
PHC	0.05 [0.00, 0.23]	0.00 [0.00, 0.01]	-1.83	-0.91	0.084
LO1	0.24 [0.19, 0.28]	0.11 [0.07, 0.19]	-3.00	-0.54	0.003
LO2	0.27 [0.24, 0.29]	0.04 [0.00, 0.15]	-4.69	-0.83	1×10^{-5}
TO1	0.34 [0.29, 0.38]	0.27 [0.19, 0.31]	-3.61	-0.64	6×10^{-4}
TO2	0.30 [0.27, 0.32]	0.32 [0.24, 0.37]	-0.06	-0.01	1
V3AB	0.20 [0.16, 0.27]	0.16 [0.10, 0.21]	-2.02	-0.38	0.057
IPS0	0.23 [0.21, 0.27]	0.22 [0.18, 0.29]	-0.67	-0.12	0.554
IPS1	0.09 [0.07, 0.15]	0.21 [0.17, 0.25]	3.22	0.57	0.002
IPS2	0.09 [0.06, 0.11]	0.23 [0.21, 0.28]	4.67	0.83	1×10^{-5}
IPS3	0.10 [0.08, 0.13]	0.27 [0.23, 0.32]	4.77	0.84	1×10^{-5}
IPS4	0.15 [0.11, 0.20]	0.24 [0.20, 0.27]	4.41	0.78	3×10^{-5}
IPS5	0.12 [0.09, 0.16]	0.24 [0.20, 0.32]	4.81	0.85	1×10^{-5}
sPCS1	0.06 [0.05, 0.08]	0.24 [0.18, 0.30]	4.94	0.87	1×10^{-5}
sPCS2	0.11 [0.09, 0.14]	0.28 [0.22, 0.32]	4.62	0.84	1×10^{-5}
iPCS	0.13 [0.12, 0.16]	0.19 [0.15, 0.24]	3.18	0.58	0.002
Threshold 0.4					
V1	0.35 [0.21, 0.46]	0.15 [0.00, 0.30]	-2.20	-0.90	0.042
V2	0.37 [0.33, 0.39]	0.20 [0.00, 0.38]	-2.49	-0.60	0.023
V3	0.37 [0.35, 0.42]	0.35 [0.31, 0.40]	-0.50	-0.10	0.758
hV4	0.39 [0.31, 0.44]	0.04 [0.00, 0.40]	-2.13	-0.59	0.046
VO1	0.21	0.25			
VO2	0.26	0.32			
PHC					
LO1	0.43 [0.40, 0.45]	0.26 [0.03, 0.38]	-2.54	-0.52	0.021
LO2	0.41 [0.37, 0.45]	0.20 [0.00, 0.33]	-3.19	-0.59	0.004
TO1	0.45 [0.40, 0.51]	0.46 [0.39, 0.50]	-1.20	-0.22	0.302
TO2	0.41 [0.37, 0.45]	0.50 [0.46, 0.54]	2.86	0.51	0.010
V3AB	0.39 [0.27, 0.46]	0.42 [0.38, 0.48]	0.12	0.03	1
IPS0	0.40 [0.33, 0.41]	0.46 [0.38, 0.49]	2.35	0.42	0.030
IPS1	0.26 [0.21, 0.32]	0.40 [0.35, 0.43]	2.68	0.54	0.016
IPS2	0.12 [0.08, 0.14]	0.41 [0.39, 0.43]	4.78	0.87	2×10^{-5}
IPS3	0.15 [0.12, 0.20]	0.43 [0.38, 0.48]	4.72	0.86	2×10^{-5}
IPS4	0.27 [0.19, 0.36]	0.48 [0.42, 0.50]	4.05	0.75	2×10^{-4}
IPS5	0.21 [0.16, 0.26]	0.44 [0.42, 0.48]	4.52	0.87	4×10^{-5}
sPCS1	0.11 [0.08, 0.17]	0.44 [0.38, 0.47]	4.29	0.87	6×10^{-5}
sPCS2	0.17 [0.14, 0.22]	0.52 [0.42, 0.55]	4.46	0.87	4×10^{-5}
iPCS	0.21 [0.15, 0.24]	0.40 [0.37, 0.42]	4.29	0.87	6×10^{-5}

Supplementary Table 1. Descriptive and test statistics of the paired model comparisons using medians per hemisphere for various variance explained thresholds in visual field maps. Data are median cross-validated variance explained [95% confidence interval of the median computed from 1000 bootstrap iterations]. These are the outcomes of a two-sided Wilcoxon signed-rank test (FDR corrected for multiple comparisons). Note that no statistical comparisons were done for VO1, VO2, and PHC under the threshold of 0.4, as in those cases $n \leq 1$.

Timing map	Monotonic fit (R^2)	Tuned fit (R^2)	*Z* statistic	Effect size (*r*)	*p* value
Threshold 0					
TLO	0.11 [0.08, 0.12]	0.00 [0.00, 0.02]	-4.92	-0.87	5×10^{-6}
TTOP	0.16 [0.14, 0.21]	0.03 [0.01, 0.07]	-4.88	-0.86	5×10^{-6}
TTOA	0.15 [0.08, 0.18]	0.05 [0.03, 0.09]	-3.44	-0.61	0.001
TPO	0.07 [0.06, 0.09]	0.05 [0.01, 0.08]	-1.87	-0.33	0.077
TLS	0.08 [0.06, 0.10]	0.04 [0.02, 0.06]	-2.89	-0.53	0.006
TPCI	0.04 [0.03, 0.05]	0.02 [0.01, 0.04]	-1.56	-0.30	0.131
TPCM	0.06 [0.05, 0.07]	0.08 [0.04, 0.09]	0.63	0.11	0.530
TPCS	0.04 [0.03, 0.05]	0.10 [0.04, 0.12]	4.30	0.76	6×10^{-5}
TFI	0.05 [0.03, 0.06]	0.02 [0.01, 0.05]	-3.30	-0.60	0.002
TFS	0.03 [0.03, 0.04]	0.06 [0.02, 0.09]	3.52	0.62	0.001
Threshold 0.2					
TLO	0.25 [0.23, 0.28]	0.15 [0.12, 0.19]	-4.06	-0.72	1×10^{-4}
TTOP	0.35 [0.29, 0.36]	0.26 [0.23, 0.33]	-2.80	-0.50	0.006
TTOA	0.28 [0.27, 0.33]	0.32 [0.24, 0.37]	0.50	0.09	0.614
TPO	0.23 [0.19, 0.26]	0.24 [0.21, 0.28]	0.62	0.11	0.597
TLS	0.24 [0.17, 0.27]	0.32 [0.23, 0.35]	3.34	0.61	0.001
TPCI	0.12 [0.09, 0.15]	0.20 [0.17, 0.26]	4.01	0.77	1×10^{-4}
TPCM	0.16 [0.10, 0.19]	0.28 [0.24, 0.33]	4.86	0.86	4×10^{-6}
TPCS	0.08 [0.07, 0.11]	0.28 [0.22, 0.31]	4.94	0.87	4×10^{-6}
TFI	0.12 [0.10, 0.15]	0.22 [0.16, 0.26]	3.44	0.65	1×10^{-3}
TFS	0.08 [0.07, 0.10]	0.26 [0.20, 0.31]	4.94	0.87	4×10^{-6}
Threshold 0.4					
TLO	0.40 [0.37, 0.43]	0.33 [0.23, 0.40]	-2.15	-0.38	0.035
TTOP	0.45 [0.41, 0.49]	0.49 [0.45, 0.52]	0.21	0.04	0.837
TTOA	0.41 [0.31, 0.44]	0.49 [0.44, 0.53]	3.86	0.70	2×10^{-4}
TPO	0.38 [0.32, 0.44]	0.45 [0.42, 0.46]	2.82	0.50	0.006
TLS	0.35 [0.25, 0.38]	0.46 [0.39, 0.53]	4.59	0.85	1×10^{-5}
TPCI	0.17 [0.10, 0.24]	0.43 [0.39, 0.45]	3.82	0.88	2×10^{-4}
TPCM	0.26 [0.19, 0.31]	0.45 [0.43, 0.48]	4.78	0.87	1×10^{-5}
TPCS	0.14 [0.11, 0.18]	0.42 [0.38, 0.46]	4.70	0.87	1×10^{-5}
TFI	0.22 [0.14, 0.28]	0.39 [0.36, 0.42]	4.03	0.82	1×10^{-4}
TFS	0.17 [0.12, 0.22]	0.46 [0.41, 0.50]	4.62	0.87	1×10^{-5}

Supplementary Table 2. Descriptive and test statistics of the paired model comparisons using medians per hemisphere for various variance explained thresholds in timing maps. Data are median cross-validated variance explained [95% confidence interval of the median computed from 1000 bootstrap iterations]. These are the outcomes of a two-sided Wilcoxon signed-rank test (FDR corrected for multiple comparisons.

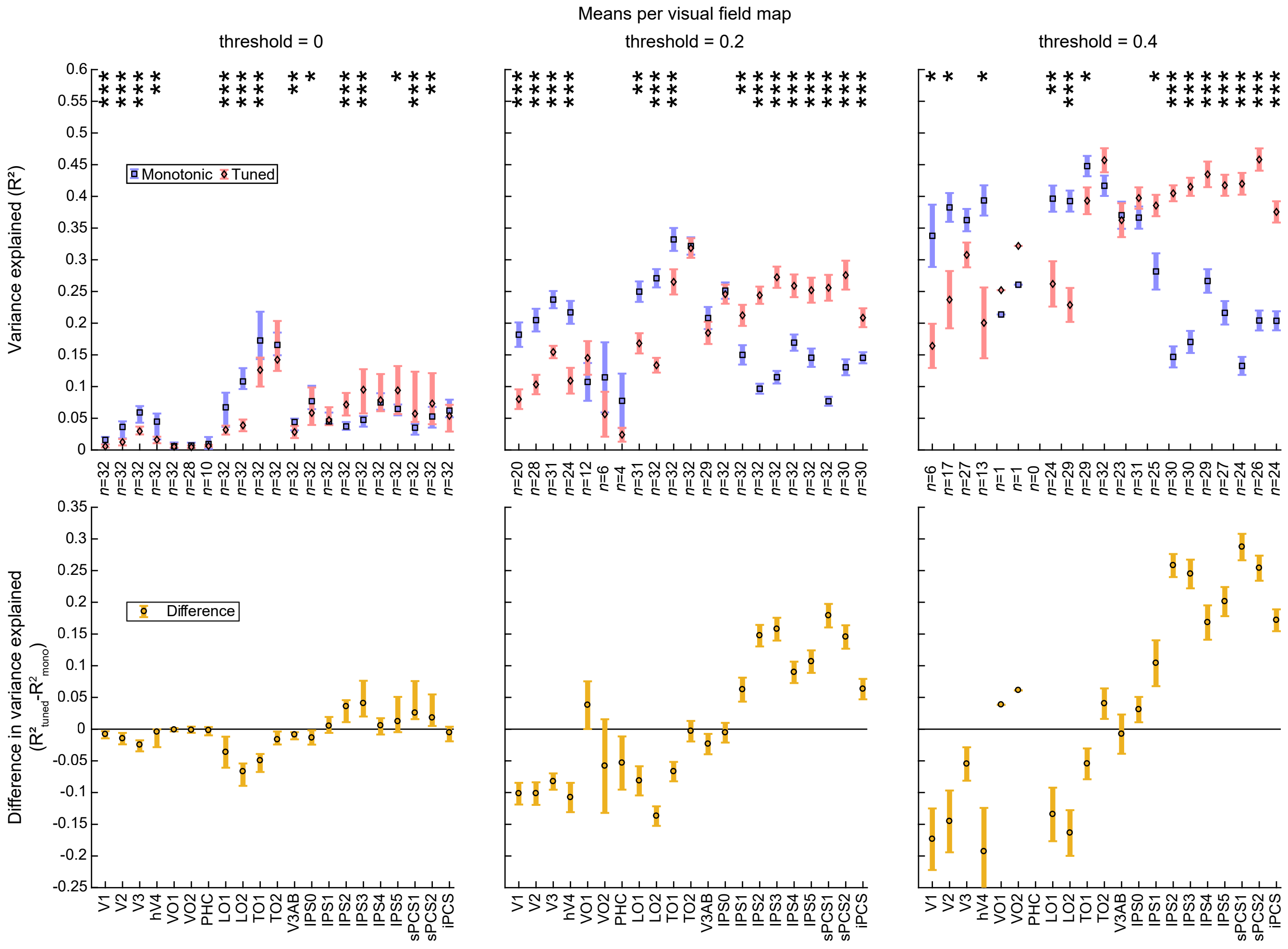

Means per visual field map
threshold = 0
threshold = 0.2
threshold = 0.4
Variance explained (R²)
Monotonic
Tuned
Difference in variance explained
(R²tuned-R²mono)
Difference
V1 V2 V3 hV4 VO1 VO2 PHC LO1 LO2 TO1 TO2 V3AB IPS0 IPS1 IPS2 IPS3 IPS4 IPS5 sPCS1 sPCS2 iPCS

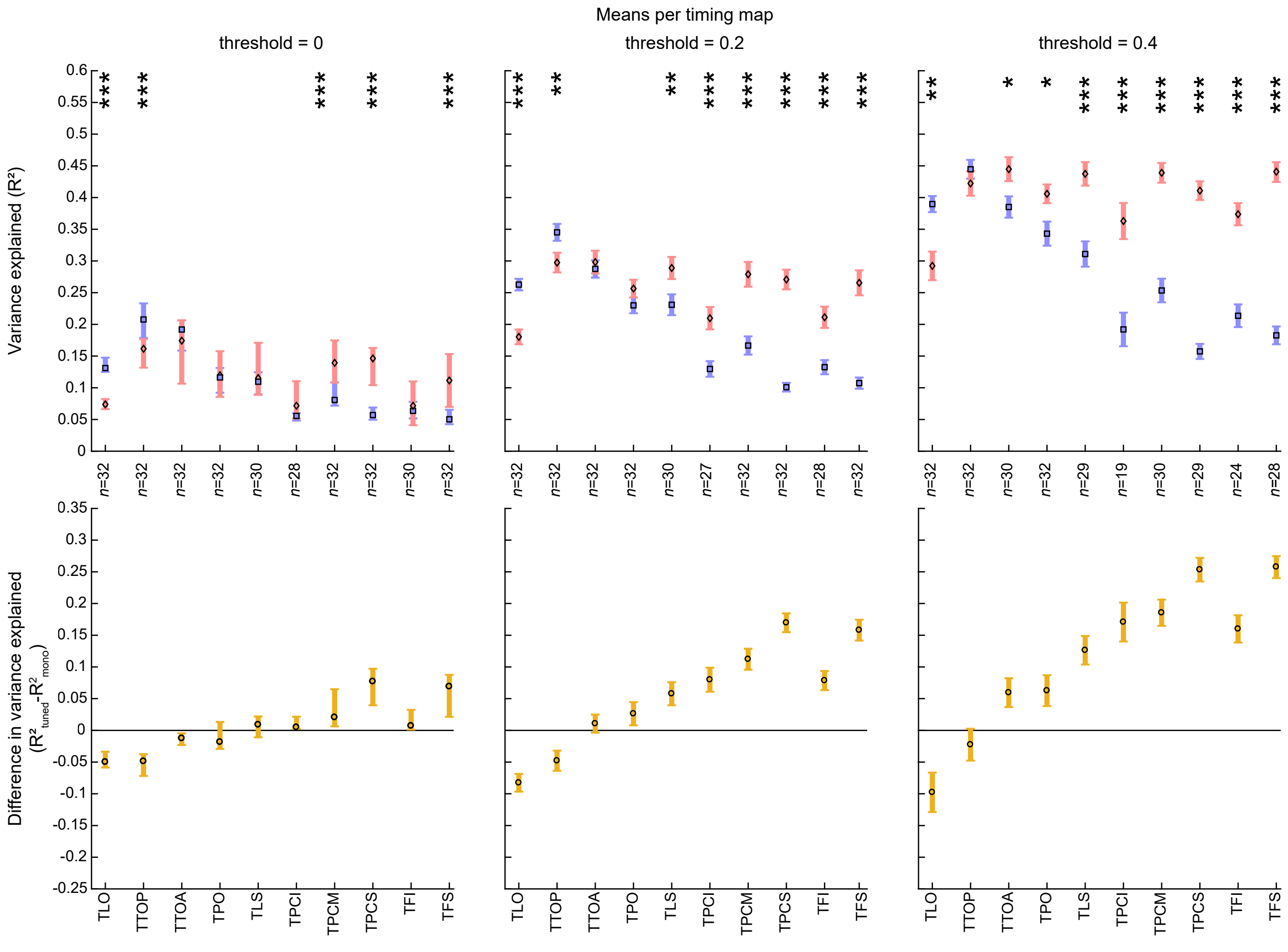

Means per timing map
threshold = 0
threshold = 0.2
threshold = 0.4
Variance explained (R²)
Difference in variance explained
(R²tuned-R²mono)
TLO TTOP TTOA TPO TLS TPCI TPCM TPCS TFI TFS

Supplementary Fig. 7: Model comparisons using means per hemisphere show similar results regardless of the variance explained threshold used for both visual field maps and timing maps. The threshold for voxel selection was a variance explained of either 0 (left column), 0.2 (middle column), or 0.4 (right column) for the best fitting model in the data on which the model was fit. All thresholds result in a gradual transition from monotonic to tuned responses, starting in similar areas. In all cases, the mean value of cross-validated variance explained was taken after thresholding in each measured visual field map example. At a threshold of 0, these means were not normally distributed (Jarque-Bera test with FDR correction), so points show the median across measured visual field map examples, error bars show 95% confidence intervals (from 1000 bootstrap iterations) and two-sided Wilcoxon signed rank tests were used. At other thresholds, the means were normally distributed (Jarque-Bera test with FDR correction), points show the mean across measured visual field map examples, error bars show the standard error of the mean, and two-sided paired t-tests were used in visual field maps where $n>1$. $^{*}p < 0.05$ $^{**}p < 0.01$, and $^{***}p < 0.001$, FDR corrected for multiple comparisons. n indicates the amount of hemispheres included in the comparison in each map. Source data are provided as a Source Data file.

Visual field map	Monotonic fit (R^2)	Tuned fit (R^2)	*Z* statistic	Effect size (*r*)	*p* value
Threshold 0					
V1	0.02 [0.01, 0.02]	0.01 [0.00, 0.01]	-4.11	-0.73	1×10^{-4}
V2	0.04 [0.02, 0.05]	0.01 [0.01, 0.02]	-4.73	-0.84	1×10^{-5}
V3	0.06 [0.04, 0.07]	0.03 [0.02, 0.04]	-4.94	-0.87	7×10^{-6}
hV4	0.04 [0.02, 0.06]	0.02 [0.01, 0.02]	-3.03	-0.54	0.004
VO1	0.01 [0.00, 0.01]	0.01 [0.00, 0.01]	-0.54	-0.10	0.588
VO2	0.01 [0.00, 0.01]	0.00 [0.00, 0.01]	-0.84	-0.16	0.419
PHC	0.01 [0.00, 0.02]	0.01 [0.00, 0.01]	-1.17	-0.37	0.281
LO1	0.07 [0.04, 0.09]	0.03 [0.02, 0.04]	-4.13	-0.73	1×10^{-4}
LO2	0.11 [0.10, 0.13]	0.04 [0.03, 0.05]	-4.94	-0.87	7×10^{-6}
TO1	0.17 [0.14, 0.22]	0.13 [0.10, 0.15]	-4.58	-0.81	2×10^{-5}
TO2	0.17 [0.15, 0.19]	0.14 [0.12, 0.20]	-1.94	-0.34	0.073
V3AB	0.04 [0.03, 0.05]	0.03 [0.02, 0.04]	-3.20	-0.57	0.003
IPS0	0.08 [0.06, 0.10]	0.06 [0.04, 0.10]	-2.62	-0.46	0.014
IPS1	0.04 [0.04, 0.06]	0.05 [0.04, 0.07]	1.61	0.28	0.142
IPS2	0.04 [0.03, 0.04]	0.07 [0.05, 0.09]	3.83	0.68	3×10^{-4}
IPS3	0.05 [0.04, 0.05]	0.10 [0.06, 0.13]	4.51	0.80	2×10^{-5}
IPS4	0.07 [0.06, 0.09]	0.08 [0.06, 0.12]	1.48	0.26	0.172
IPS5	0.06 [0.05, 0.07]	0.09 [0.06, 0.13]	2.51	0.44	0.018
sPCS1	0.04 [0.02, 0.04]	0.06 [0.04, 0.12]	4.88	0.86	7×10^{-6}
sPCS2	0.05 [0.04, 0.07]	0.07 [0.04, 0.12]	3.38	0.60	0.001
iPCS	0.06 [0.05, 0.08]	0.05 [0.03, 0.07]	-0.86	-0.15	0.419

Supplementary Table 3. Descriptive and test statistics of the paired model comparisons using means per hemisphere for a variance explained threshold of 0 in visual field maps. Data are median cross-validated variance explained [95% confidence interval of the median computed from 1000 bootstrap iterations]. These are the outcomes of a two-sided Wilcoxon signed-rank test (FDR corrected for multiple comparisons).

Visual field map	Monotonic fit (R^2)	Tuned fit (R^2)	*t* statistic	Effect size (*d'*)	*p* value
Threshold 0.2					
V1	0.18 [0.16, 0.20]	0.08 [0.06, 0.10]	-5.94	-1.33	2×10^{-5}
V2	0.20 [0.19, 0.22]	0.10 [0.09, 0.12]	-5.70	-1.08	1×10^{-5}
V3	0.24 [0.22, 0.25]	0.15 [0.14, 0.16]	-6.41	-1.15	2×10^{-6}
hV4	0.22 [0.20, 0.24]	0.11 [0.09, 0.13]	-4.66	-0.95	2×10^{-4}
VO1	0.11 [0.08, 0.14]	0.15 [0.12, 0.17]	1.01	0.29	0.392
VO2	0.11 [0.06, 0.17]	0.06 [0.02, 0.09]	-0.79	-0.32	0.515
PHC	0.08 [0.03, 0.12]	0.02 [0.01, 0.04]	-1.27	-0.64	0.362
LO1	0.25 [0.23, 0.27]	0.17 [0.15, 0.18]	-3.54	-0.64	0.002
LO2	0.27 [0.26, 0.29]	0.13 [0.12, 0.15]	-8.90	-1.57	5×10^{-9}
TO1	0.33 [0.31, 0.35]	0.27 [0.25, 0.29]	-4.37	-0.77	2×10^{-4}
TO2	0.32 [0.31, 0.34]	0.32 [0.30, 0.33]	-0.20	-0.04	0.843
V3AB	0.21 [0.19, 0.23]	0.18 [0.17, 0.20]	-1.46	-0.27	0.203
IPS0	0.25 [0.24, 0.26]	0.25 [0.23, 0.26]	-0.36	-0.06	0.756
IPS1	0.15 [0.13, 0.17]	0.21 [0.20, 0.23]	3.29	0.58	0.003
IPS2	0.10 [0.09, 0.10]	0.24 [0.23, 0.26]	8.67	1.53	5×10^{-9}
IPS3	0.11 [0.10, 0.12]	0.27 [0.26, 0.29]	8.77	1.55	5×10^{-9}
IPS4	0.17 [0.16, 0.18]	0.26 [0.24, 0.28]	5.30	0.94	2×10^{-5}
IPS5	0.15 [0.13, 0.16]	0.25 [0.23, 0.27]	6.00	1.06	4×10^{-6}
sPCS1	0.08 [0.07, 0.08]	0.26 [0.24, 0.28]	9.64	1.70	2×10^{-9}
sPCS2	0.13 [0.12, 0.14]	0.28 [0.25, 0.30]	7.87	1.44	5×10^{-8}
iPCS	0.15 [0.14, 0.15]	0.21 [0.19, 0.22]	3.92	0.72	8×10^{-4}
Threshold 0.4					
V1	0.34 [0.29, 0.39]	0.16 [0.13, 0.20]	-3.58	-1.46	0.026
V2	0.38 [0.36, 0.41]	0.24 [0.19, 0.28]	-2.99	-0.72	0.017
V3	0.36 [0.34, 0.38]	0.31 [0.29, 0.33]	-2.09	-0.40	0.066
hV4	0.39 [0.37, 0.42]	0.20 [0.14, 0.26]	-2.80	-0.78	0.026
VO1	0.21	0.25			
VO2	0.26	0.32			
PHC					
LO1	0.40 [0.38, 0.42]	0.26 [0.23, 0.30]	-3.19	-0.65	0.010
LO2	0.39 [0.38, 0.41]	0.23 [0.20, 0.26]	-4.54	-0.84	3×10^{-4}
TO1	0.45 [0.43, 0.46]	0.39 [0.37, 0.41]	-2.25	-0.42	0.049
TO2	0.42 [0.40, 0.43]	0.46 [0.44, 0.48]	1.67	0.30	0.137
V3AB	0.37 [0.35, 0.39]	0.36 [0.34, 0.39]	-0.25	-0.05	0.936
IPS0	0.37 [0.35, 0.38]	0.40 [0.38, 0.41]	1.54	0.28	0.165
IPS1	0.28 [0.25, 0.31]	0.39 [0.37, 0.40]	2.87	0.57	0.017
IPS2	0.15 [0.13, 0.16]	0.40 [0.39, 0.42]	14.19	2.59	3×10^{-13}
IPS3	0.17 [0.15, 0.19]	0.42 [0.40, 0.43]	10.81	1.97	6×10^{-11}
IPS4	0.27 [0.25, 0.29]	0.43 [0.41, 0.45]	6.21	1.15	3×10^{-6}
IPS5	0.22 [0.20, 0.23]	0.42 [0.40, 0.43]	8.75	1.68	1×10^{-8}
sPCS1	0.13 [0.12, 0.15]	0.42 [0.40, 0.44]	13.77	2.81	1×10^{-11}
sPCS2	0.20 [0.19, 0.22]	0.46 [0.44, 0.48]	12.80	2.51	1×10^{-11}
iPCS	0.20 [0.19, 0.22]	0.38 [0.36, 0.39]	9.96	2.03	3×10^{-9}

Supplementary Table 4. Descriptive and test statistics of the paired model comparisons using means per hemisphere for a variance explained threshold of 0.2 and 0.4 in visual field maps. Data are mean cross-validated variance explained [+/- standard error of the mean]. These are the outcomes of a two-sided paired t test (FDR corrected for multiple comparisons) where effect size is calculated as $d' = t / \sqrt{n}$. Note that no statistical comparisons were done for VO1, VO2, and PHC under the threshold of 0.4, as in those cases $n \leq 1$.

Timing map	Monotonic fit (R^2)	Tuned fit (R^2)	*Z* statistic	Effect size (*r*)	*p* value
Threshold 0					
TLO	0.13 [0.12, 0.15]	0.07 [0.07, 0.08]	-4.77	-0.84	6×10^{-6}
TTOP	0.21 [0.18, 0.23]	0.16 [0.13, 0.18]	-3.55	-0.63	8×10^{-4}
TTOA	0.19 [0.16, 0.21]	0.17 [0.11, 0.21]	-1.38	-0.24	0.208
TPO	0.12 [0.09, 0.13]	0.12 [0.09, 0.16]	-0.30	-0.05	0.765
TLS	0.11 [0.09, 0.12]	0.12 [0.09, 0.17]	1.29	0.23	0.221
TPCI	0.06 [0.05, 0.06]	0.07 [0.05, 0.11]	2.16	0.41	0.051
TPCM	0.08 [0.07, 0.11]	0.14 [0.11, 0.17]	3.80	0.67	4×10^{-4}
TPCS	0.06 [0.05, 0.07]	0.15 [0.10, 0.16]	4.92	0.87	6×10^{-6}
TFI	0.06 [0.05, 0.08]	0.07 [0.04, 0.11]	1.98	0.36	0.067
TFS	0.05 [0.04, 0.07]	0.11 [0.07, 0.15]	4.82	0.85	6×10^{-6}

Supplementary Table 5. Descriptive and test statistics of the paired model comparisons using means per hemisphere for a threshold of 0 variance explained in timing maps. Data are median cross-validated variance explained [95% confidence interval of the median computed from 1000 bootstrap iterations]. These are the outcomes of a two-sided Wilcoxon signed-rank test (FDR corrected for multiple comparisons).

Timing map	Monotonic fit (R^2)	Tuned fit (R^2)	*t* statistic	Effect size (*d'*)	*p* value
Threshold 0.2					
TLO	0.26 [0.25, 0.27]	0.18 [0.17, 0.19]	-5.89	-1.04	4×10^{-6}
TTOP	0.35 [0.33, 0.36]	0.30 [0.28, 0.31]	-2.99	-0.53	0.007
TTOA	0.29 [0.27, 0.30]	0.30 [0.28, 0.32]	0.76	0.13	0.453
TPO	0.23 [0.22, 0.24]	0.26 [0.24, 0.27]	1.44	0.25	0.177
TLS	0.23 [0.21, 0.25]	0.29 [0.27, 0.31]	3.18	0.58	0.005
TPCI	0.13 [0.12, 0.14]	0.21 [0.19, 0.23]	4.22	0.81	4×10^{-4}
TPCM	0.17 [0.15, 0.18]	0.28 [0.26, 0.30]	6.79	1.20	4×10^{-7}
TPCS	0.10 [0.09, 0.11]	0.27 [0.26, 0.29]	11.44	2.02	1×10^{-11}
TFI	0.13 [0.12, 0.14]	0.21 [0.19, 0.23]	5.23	0.99	3×10^{-5}
TFS	0.11 [0.10, 0.12]	0.27 [0.25, 0.29]	9.60	1.70	4×10^{-10}
Threshold 0.4					
TLO	0.39 [0.38, 0.40]	0.29 [0.27, 0.31]	-3.14	-0.55	0.005
TTOP	0.44 [0.43, 0.46]	0.42 [0.40, 0.44]	-0.89	-0.16	0.380
TTOA	0.39 [0.37, 0.40]	0.44 [0.43, 0.46]	2.62	0.48	0.016
TPO	0.34 [0.32, 0.36]	0.41 [0.39, 0.42]	2.58	0.46	0.016
TLS	0.31 [0.29, 0.33]	0.44 [0.42, 0.46]	5.61	1.04	1×10^{-5}
TPCI	0.19 [0.17, 0.22]	0.36 [0.33, 0.39]	5.58	1.28	4×10^{-5}
TPCM	0.25 [0.23, 0.27]	0.44 [0.42, 0.45]	8.96	1.64	2×10^{-9}
TPCS	0.16 [0.15, 0.17]	0.41 [0.40, 0.43]	13.62	2.53	4×10^{-13}
TFI	0.21 [0.20, 0.23]	0.37 [0.36, 0.39]	7.45	1.52	4×10^{-7}
TFS	0.18 [0.17, 0.20]	0.44 [0.42, 0.46]	14.85	2.81	2×10^{-13}

Supplementary Table 6. Descriptive and test statistics of the paired model comparisons using means per hemisphere for a variance explained threshold of 0.2 and 0.4 in timing maps. Data are mean cross-validated variance explained [+/- standard error of the mean]. These are the outcomes of a two-sided paired *t* test (FDR corrected for multiple comparisons), where effect size is calculated as $d' = t/\sqrt{n}$.

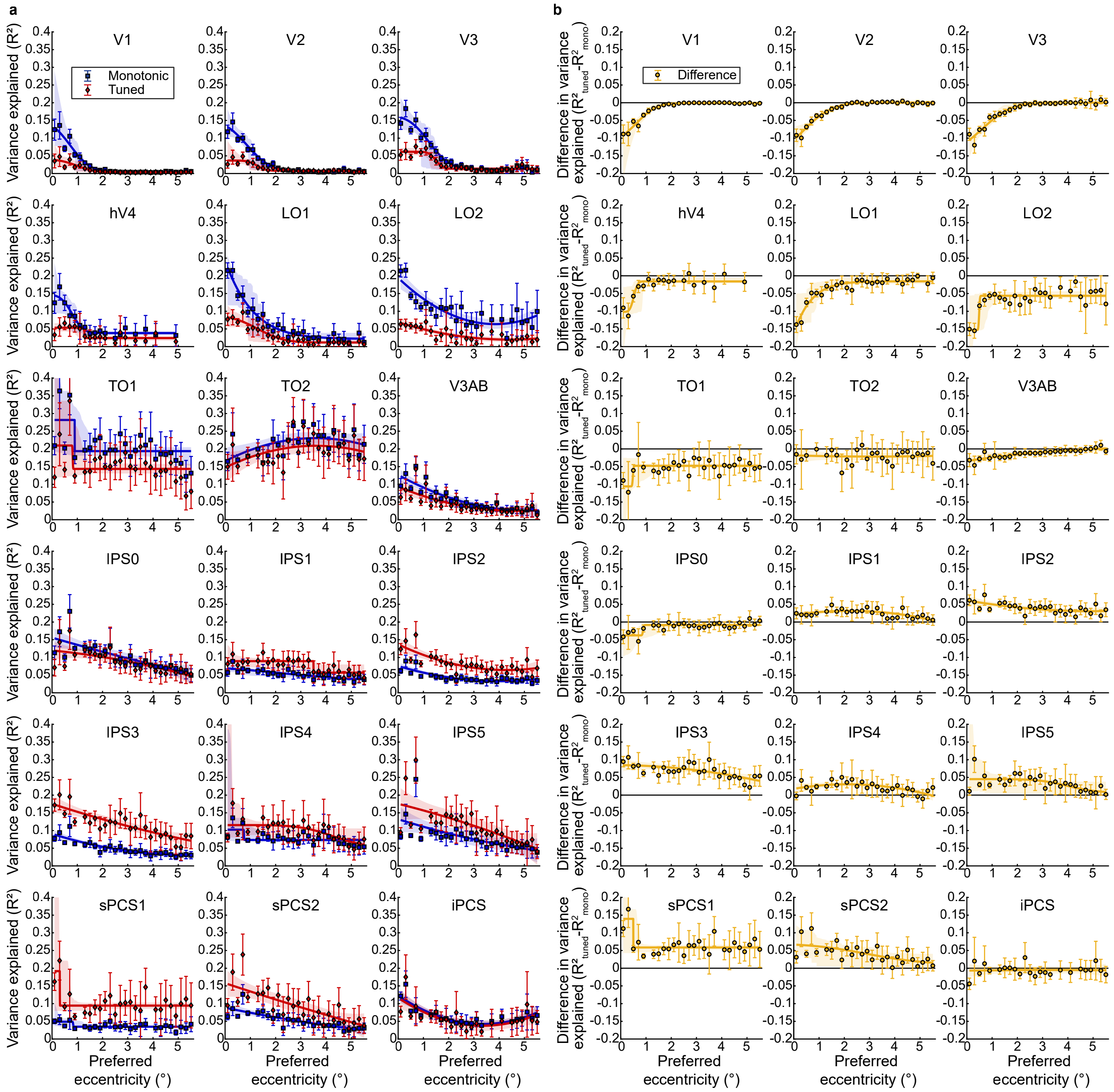

Supplementary Fig. 8 Progression of timing response model fits with preferred visual field eccentricity in all visual field maps. a Early and lateral occipital visual field maps show a sharp decrease of model fits moving away from the retinotopic representation of the stimulus position. This decrease then becomes more gradual where tuned response model fits begin to improve. **b** The difference between the response model fits (tuned - monotonic) also decreases with eccentricity in the early and lateral occipital visual field maps, but shows no consistent relationship with eccentricity after TO1. Markers show mean variance explained per eccentricity bin, error bars show the standard error of the mean. For all bins, $n \geq 50$ included voxels. Solid lines show the best fit to changes with eccentricity, shaded areas around these lines are their 95% confidence intervals. Note that the data for these plots are not thresholded at a variance explained above 0.2, but above 0 for the best fitting model in the data on which the models were fit. Source data are provided as a Source Data file.
