# Supplementary material for: Visual timing-tuned responses in human association cortices and response dynamics in early visual cortex
Source: Nat Commun. 2022 Jul 8;13:3952. doi: 10.1038/s41467-022-31675-9 (PMC9270326; doi:10.1038/s41467-022-31675-9)
Supplement: Supplementary file 3 — Description of Additional Supplementary Files [file 41467_2022_31675_MOESM3_ESM.pdf]

Description of Additional Supplementary Files

Supplementary Movie 1. Event timing stimuli in each stimulus configuration. Text and distance markers were not shown, and stimulus configuration blocks were shown in a pseudo-random order differing between scan runs. Adapted from Harvey, Dumoulin, Fracasso & Paul (2020, Current Biology).
