# Supplementary material for: Visual timing-tuned responses in human association cortices and response dynamics in early visual cortex
Source: Nat Commun. 2022 Jul 8;13:3952. doi: 10.1038/s41467-022-31675-9 (PMC9270326; doi:10.1038/s41467-022-31675-9)
Supplement: Supplementary file 5 — Reporting Summary [file 41467_2022_31675_MOESM5_ESM.pdf]

nature portfolio

Corresponding author(s): Ben Harvey
Last updated by author(s): Apr 9, 2022

Reporting Summary

Nature Portfolio wishes to improve the reproducibility of the work that we publish. This form provides structure for consistency and transparency in reporting. For further information on Nature Portfolio policies, see our Editorial Policies and the Editorial Policy Checklist.

Statistics

For all statistical analyses, confirm that the following items are present in the figure legend, table legend, main text, or Methods section.

n/a	Confirmed	
☐	☒	The exact sample size (*n*) for each experimental group/condition, given as a discrete number and unit of measurement
☐	☒	A statement on whether measurements were taken from distinct samples or whether the same sample was measured repeatedly
☐	☒	The statistical test(s) used AND whether they are one- or two-sided<br>*Only common tests should be described solely by name; describe more complex techniques in the Methods section.*
☐	☒	A description of all covariates tested
☐	☒	A description of any assumptions or corrections, such as tests of normality and adjustment for multiple comparisons
☐	☒	A full description of the statistical parameters including central tendency (e.g. means) or other basic estimates (e.g. regression coefficient) AND variation (e.g. standard deviation) or associated estimates of uncertainty (e.g. confidence intervals)
☐	☒	For null hypothesis testing, the test statistic (e.g. F, t, r) with confidence intervals, effect sizes, degrees of freedom and P value noted<br>*Give P values as exact values whenever suitable.*
☐	☒	For Bayesian analysis, information on the choice of priors and Markov chain Monte Carlo settings
☒	☐	For hierarchical and complex designs, identification of the appropriate level for tests and full reporting of outcomes
☐	☒	Estimates of effect sizes (e.g. Cohen's *d*, Pearson's *r*), indicating how they were calculated

Our web collection on statistics for biologists contains articles on many of the points above.

Software and code

Policy information about availability of computer code

Data collection	7T Philips Achieva scanner, PsychToolbox-3, in-house IDL software (v6.3, RSI, Boulder, CO, USA)
Data analysis	For initial data (pre-)processing:<br>mrVista 2.0 (https://github.com/vistalab/vistasoft)<br>Matlab version 2018b (The Mathworks)<br>Freesurfer 6.0 (https://surfer.nmr.mgh.harvard.edu/)<br>ITK-SNAP 1.6.0.1<br>AFNI 19.1.21 (afni.nimh.nih.gov)<br><br>For later data analyses:<br>Matlab version 2017a (The Mathworks)<br>JASP 0.14.1.0 (https://jasp-stats.org)<br><br>The code that supports the findings of this study is available from the following repositories: vistasoft (github.com/vistalab/vistasoft, https://doi.org/10.5281/zenodo.5811120); vistasoftAddOns (github.com/benharvey/vistasoftAddOns, https://doi.org/10.5281/zenodo.5811114); fMRI_preproc (github.com/MvaOosterhuis/fMRI_preproc, https://doi.org/10.5281/zenodo.5811116); MonoTunedTiming (github.com/evi-hendrikx/MonoTunedTiming, https://doi.org/10.5281/zenodo.6417921).

For manuscripts utilizing custom algorithms or software that are central to the research but not yet described in published literature, software must be made available to editors and reviewers. We strongly encourage code deposition in a community repository (e.g. GitHub). See the Nature Portfolio guidelines for submitting code & software for further information.

Data

Policy information about availability of data

All manuscripts must include a data availability statement. This statement should provide the following information, where applicable:

- Accession codes, unique identifiers, or web links for publicly available datasets
- A description of any restrictions on data availability
- For clinical datasets or third party data, please ensure that the statement adheres to our policy

Ethical constraints prevent us from sharing the medical imaging data sets (MRI scans) generated in the current study to public repositories. The structure of the brain is unique to the individual participant, in theory allowing the participant to be identified from these images, which may also contain medically sensitive findings. This is an interpretation of the EU's General Data Protection Regulation (GDPR) for medical images including MRI data. These raw data sets are available from the corresponding author upon reasonable request within a month, depending on agreements not to share these data publicly. Model parameters underlying all statistical analyses and response data for all model fitting are publicly available at the following DOIs: visual field map parameters (doi.org/10.6084/m9.figshare.19146131), timing map parameters (doi.org/10.6084/m9.figshare.17122706), parameters used during validation (doi.org/10.6084/m9.figshare.17122727), visual field map time series (doi.org/10.6084/m9.figshare.19146092), timing map time series (doi.org/10.6084/m9.figshare.17122718) and validation time series (doi.org/10.6084/m9.figshare.17122748). Source Data plotted in the Figures are provided with this paper.

Field-specific reporting

Please select the one below that is the best fit for your research. If you are not sure, read the appropriate sections before making your selection.

☐ Life sciences ☒ Behavioural & social sciences ☐ Ecological, evolutionary & environmental sciences

For a reference copy of the document with all sections, see nature.com/documents/nr-reporting-summary-flat.pdf

Behavioural & social sciences study design

All studies must disclose on these points even when the disclosure is negative.

Study description	Here we ask whether and how monotonic and tuned neural responses to sub-second visual event timing are related throughout the brain's hierarchy of both timing maps and visual field maps. We further ask how both monotonic and tuned responses to timing are related to visual position preferences. We answer these questions by reanalyzing ultra-high-field (7T) functional magnetic resonance imaging (fMRI) data that was acquired during the presentation of repetitive visual events that gradually varied in event duration and/or period in our previous study (Harvey et al., 2020) (quantitative). We compare the fits of monotonically increasing and tuned neural response models throughout the brain and investigate how these relate to visual spatial selectivity throughout the visual field map hierarchy.
Research sample	We acquired fMRI data from eight participants (1 female, aged 25 to 35). All participants were right-handed and had normal or corrected to normal vision. The rationale for selecting a research sample of healthy neurotypical participants is that we were interested in studying normal brain function. As we do not expect differences in the representations of timing in the brain between sexes, age, or educational level, we consider this sample representative.
Sampling strategy	We used a convenience sample of 8 researchers. Two participants were co-authors, familiar with the goals of the study. The remaining six were graduate students from elsewhere in our university and hospital recruited through advertising.<br>No sample size calculation was performed, as all data was originally collected for another study (Harvey et al., 2020, Current Biology). In this other study, sample sizes were chosen based on the sample sizes that had previously been used to convincingly demonstrate reproducibility in experiments with similar designs throughout the field.
Data collection	We acquired MRI data on a 7T Philips Achieva scanner.<br><br>No one else was present besides the participant(s) and the researchers during data collection. This data was originally collected for another study, where the operator was aware of the hypothesis of that study. This hypothesis entailed the existence of topographic maps for timing. In the experimental setup there were no experimental conditions that were contrasted to each other, so the researchers were not blinded.
Timing	Data were collected from 21/02/2016 to 10/04/2018.
Data exclusions	Data from all scanned participants was included. However, we acquired data from the whole brain, although only a small set of regions was analyzed, as in all fMRI experiments. Most locations in the brain do not respond to the changes in subsecond timing. We first excluded from analysis any recording sites (voxels) that lay outside the gray matter. The models were fit on all included, gray matter voxels. For further analyses where model fits were compared (except the surface renderings), voxels outside of timing maps or visual field maps were excluded. For all model comparisons, we also excluded voxels where the variance explained of both models on cross-validated data was 0.2 or less. For other further analyses where eccentricity ranges were compared, voxels outside of visual field maps or with a preferred eccentricity above 5.6 degrees were excluded. Of this subset, we also excluded voxels that did not respond to timing (variance explained of 0 for both models). This was in total several million excluded voxels.
Non-participation	No participants dropped out/declined participation

Randomization	Participants were not allocated into experimental groups

Reporting for specific materials, systems and methods

We require information from authors about some types of materials, experimental systems and methods used in many studies. Here, indicate whether each material, system or method listed is relevant to your study. If you are not sure if a list item applies to your research, read the appropriate section before selecting a response.

Materials & experimental systems

n/a	Involved in the study
☒	☐ Antibodies
☒	☐ Eukaryotic cell lines
☒	☐ Palaeontology and archaeology
☒	☐ Animals and other organisms
☐	☒ Human research participants
☒	☐ Clinical data
☒	☐ Dual use research of concern

Methods

n/a	Involved in the study
☒	☐ ChIP-seq
☒	☐ Flow cytometry
☐	☒ MRI-based neuroimaging

Human research participants

Policy information about studies involving human research participants

Population characteristics	See above
Recruitment	Participants were recruited from Utrecht University and Utrecht Medical Center either in response to recruitment posters for the study or from the lab. We do not expect any biases as a result from our recruitment method to influence our results, as we expect timing representations in the brain to be present in all individuals
Ethics oversight	All experimental procedures were approved by the ethics committee of University Medical Center Utrecht

Note that full information on the approval of the study protocol must also be provided in the manuscript.

Magnetic resonance imaging

Experimental design

Design type	Task fMRI
Design specifications	FMRI was acquired during presentation of repetitive visual events which gradually varied in event duration and/or period in the range of 50-1000 ms with 50 ms steps. The visual events comprised the presentation of a filled circle with a diameter of 0.4° that appeared for a variable duration at a variable temporal period. The position of the circle changed pseudo-randomly between stimulus events.<br>Each event timing was repeated in a 2100 ms time frame, such that an entire TR (=2100 ms) contained the same event duration and period. The number of events presented within the 2100 ms between timing changes varied with event period and increments in event period sometimes fell slightly before or after 2100 ms. The maximum drift of event onset timing was only 300 ms, and the increments in event period were only 50 ms, so this deviation was not perceptible. The presented event timing was used for analysis.<br>There were 4 we used four conditions where event duration and period were related in different ways. In all conditions, increasing progressions of event duration and/or period were followed by an interval of events with a 2100 ms period (16.8 s or, when there were two ranges: 6.3 s). The event duration here was 50 or 2000 ms, depending on the condition. These extreme timings help to distinguish very small and very large response functions in fMRI responses. Then, the same event timings were presented in a decreasing order, again followed by interval of events with a 50 or 2000 ms duration and a 2100 ms period (16.8 s or, when there were two ranges: 6.3 s and 14.7 s). We tested each of 24 possible orders of all four stimulus configurations once per participant, so each participant's data included 24 scanning runs, each totaling 470.4 seconds and acquired in four sessions. In each scan session, we also acquired a top-up scan with the opposite phase-encoding direction to correct for image distortion in the gradient encoding direction, and a T1-weighted anatomical image with the same resolution, position and orientation as the functional data.<br>For visual field mapping, a bar filled with a moving checkerboard pattern stepped across a 6.35° (radius) circle in the display center in eight (cardinal and diagonal) directions.
Behavioral performance measures	During visual field mapping the central fixation cross changed color (red/green) and during presentation of visual event time stimuli the dot changed color (black/white) at pseudo-random intervals (on average once every 21 seconds). Participants were instructed to press a button when a color change occurred to confirm they were paying attention to the stimuli and remained awake throughout scanning.

Acquisition

Imaging type(s)	Functional
Field strength	7 Tesla
Sequence & imaging parameters	We acquired T1-weighted anatomical scans and T2*-weighted functional images using a 32-channel head coil at a resolution of 1.77×1.77×1.75 mm, with 41 interleaved slices of 128×128 voxels. The resulting field of view was 227×227×72 mm. TR was 2100 ms, TE was 25 ms, and flip angle was 70 degrees. We used a single shot gradient echo sequence with SENSE acceleration factor 3.0 and anterior-posterior encoding.
Area of acquisition	Whole brain scan (excluding anterior frontal and temporal lobes, where 7T fMRI has low response amplitudes and large spatial distortions)

Diffusion MRI ☐ Used ☒ Not used

Preprocessing

Preprocessing software	Freesurfer 6.0 (https://surfer.nmr.mgh.harvard.edu/), ITK-SNAP 1.6.0.1, AFNI 19.1.21 (afni.nimh.nih.gov)
Normalization	Analyses were performed in each participants' native space
Normalization template	Analyses were performed in each participants' native space
Noise and artifact removal	Functional scans were corrected for head movement and motion with two series of images that were acquired using opposing phase-encoding directions, with transformations calculated using AFNI (3dvolreg, 3dQwarp, 3dNwarpApply). No other spatial or temporal smoothing procedures were applied
Volume censoring	There was no volume censoring

Statistical modeling & inference

Model type and settings	Population receptive field modeling and general linear models
Effect(s) tested	Model fits with variance explained

Specify type of analysis: ☒ Whole brain ☐ ROI-based ☐ Both

Statistic type for inference (See Eklund et al. 2016)	Voxel-wise
Correction	The voxel-wise population receptive field model and monotonic response model fits were not corrected for multiple comparisons, but all following statistics on visual field maps and timing maps were

Models & analysis

n/a	Involved in the study
☒	☐ Functional and/or effective connectivity
☒	☐ Graph analysis
☐	☒ Multivariate modeling or predictive analysis

Multivariate modeling and predictive analysis	Population receptive field modeling and general linear models
